# Preferential binding of allosteric modulators to active and inactive conformational states of metabotropic glutamate receptors

**DOI:** 10.1186/1471-2105-9-S1-S16

**Published:** 2008-02-13

**Authors:** Naveena Yanamala, Kalyan C Tirupula, Judith Klein-Seetharaman

**Affiliations:** 1 Department of Structural Biology, University of Pittsburgh School of Medicine, Pittsburgh, PA 15260, USA

## Abstract

Metabotropic glutamate receptors (mGluRs) are G protein coupled receptors that play important roles in synaptic plasticity and other neuro-physiological and pathological processes. Allosteric mGluR ligands are particularly promising drug targets because of their modulatory effects – enhancing or suppressing the response of mGluRs to glutamate. The mechanism by which this modulation occurs is not known. Here, we propose the hypothesis that positive and negative modulators will differentially stabilize the active and inactive conformations of the receptors, respectively. To test this hypothesis, we have generated computational models of the transmembrane regions of different mGluR subtypes in two different conformations. The inactive conformation was modeled using the crystal structure of the inactive, dark state of rhodopsin as template and the active conformation was created based on a recent model of the light-activated state of rhodopsin. Ligands for which the nature of their allosteric effects on mGluRs is experimentally known were docked to the modeled mGluR structures using ArgusLab and Autodock softwares. We find that the allosteric ligand binding pockets of mGluRs are overlapping with the retinal binding pocket of rhodopsin, and that ligands have strong preferences for the active and inactive states depending on their modulatory nature. In 8 out of 14 cases (57%), the negative modulators bound the inactive conformations with significant preference using both docking programs, and 6 out of 9 cases (67%), the positive modulators bound the active conformations. Considering results by the individual programs only, even higher correlations were observed: 12/14 (86%) and 8/9 (89%) for ArgusLab and 10/14 (71%) and 7/9 (78%) for AutoDock. These findings strongly support the hypothesis that mGluR allosteric modulation occurs via stabilization of different conformations analogous to those identified in rhodopsin where they are induced by photochemical isomerization of the retinal ligand – despite the extensive differences in sequences between mGluRs and rhodopsin.

## Background

Glutamate is the most important excitatory neurotransmitter in the brain. Glutamatergic neurotransmission proceeds primarily via ion gated channels (ionotropic glutamate receptors). In addition, there are metabotropic glutamate receptors (mGluRs), which belong to the G protein coupled receptor (GPCR) family and play modulatory roles in neuronal processes such as anxiety, learning, memory and perception of pain [1]. Because of these roles they form attractive drug targets for treatment of neuronal dysfunction including seizures, epilepsy, Parkinson's disease and night blindness [2-4]. Both types of glutamate receptors share a common extracellular ligand binding architecture, albeit different topology and protein family membership. X-ray crystallographic structures are available for the soluble extracellular domains of mGluRs [5-8] and ionotropic glutamate receptors [9-11]. Because of the common ligand recognized by these extracellular domains, targeting mGluRs specifically without interfering with the ubiquitous glutamatergic neurotransmission therefore requires designing allosteric ligands that bind in the transmembrane domains of mGluRs. Being members of the GPCR superfamily, mGluRs are structurally characterized by seven transmembrane helices that divide each protein into an extracellular, cytoplasmic and transmembrane domain. Based on sequence homology and pharmacological considerations the GPCR family is subdivided into several classes. Class A, the largest subfamily, is the rhodopsin like family. Members of other GPCR classes share very little sequence homology with class A members and some also differ in the length of their N-termini. In particular, the ligand binding domain of class C GPCRs is located in a 500 amino acid long addition to the N-terminus. This is unusual for the GPCR family where the ligand binding domain is typically located in the transmembrane domain, near its interface with the extracellular domain. The prototypical members of class C GPCRs are the mGluRs. In human, there are eight subtypes, divided into three groups based on their pharmacological and signaling properties. Group I mGluRs (subtypes 1 and 5) are primarily localized postsynaptically where they modulate ion channel activity and neuronal excitability. Groups II (subtypes 2 and 3) and III (subtypes 4, 6, 7, and 8) are primarily located presynaptically and regulate the release of neurotransmitters, including glutamate [12].

For Group I mGluRs it was shown by mutagenesis that allosteric modulators bind in the transmembrane domain near the interface between the transmembrane and extracellular domains similar to the ligand binding pockets in class A GPCRs [13,14]. Allosteric mGluR ligands act as positive or negative modulators of mGluR activity in response to glutamate or glutamate analogs, enhancing or suppressing the responses respectively [15]. Small changes in the chemical structures of ligands can switch their modulatory effects. For example, 4,4'-difluorobenzaldazine (4,4'-DFB) is a negative modulator for mGluR5, while 3,3'-difluorobenzaldazine (3,3'-DFB) is a positive modulator for the same receptor [16]. Being able to predict if a given ligand will have positive or negative modulatory effects on mGluRs would be highly beneficial in the design of future drug candidates targeted at these receptors. With the long-term goal of building such a predictor, we here propose the hypothesis that positive and negative modulators can be distinguished by their higher affinities for the active and inactive conformations of the receptors, respectively. To test this hypothesis, we have generated computational models of the transmembrane regions of different mGluR subtypes in two different conformations. The inactive conformation was modeled using the crystal structure of the inactive, dark state of rhodopsin as template [17] and the active conformation was created based on a recent model of the light-activated state of rhodopsin [18]. We find that in the majority of ligand-receptor pairs, binding energies for positive modulators are more favorable when docked to the active conformation than the inactive conformation and *vice versa *for negative modulators.

## Results

### Identification and analysis of the ligand binding pocket

**Figure 1:**
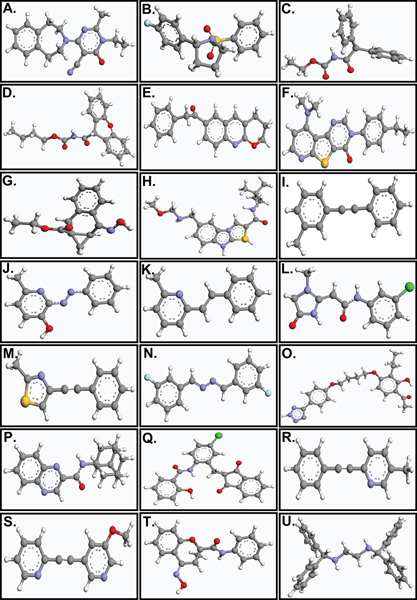
The structures of the ligands studied. (A) EM-TBPC (B) Ro67-7476 (C) Ro01-6128 (D) Ro67-4853 (E) R214127 (F) triazafluorenone (G) CPCCOEt (H) YM298198 (I) MPEP (J) SIB-1757 (K) SIB-1893 (L) Fenobam (M) MTEP (N) DFB-derivatives. The positions of the fluorine atoms are indicated for DFB-2,2' and DFB-4,4'. DFB-3,3' is shown. (O) PTEB (P) NPS2390 (Q) CPPHA (R) 5MPEP (S) MPEPy (T) PHCCC (U) AMN082. For definition of ligand names, see abbreviations list. Images were created using ArgusLab software [58].

**Figure 2:**
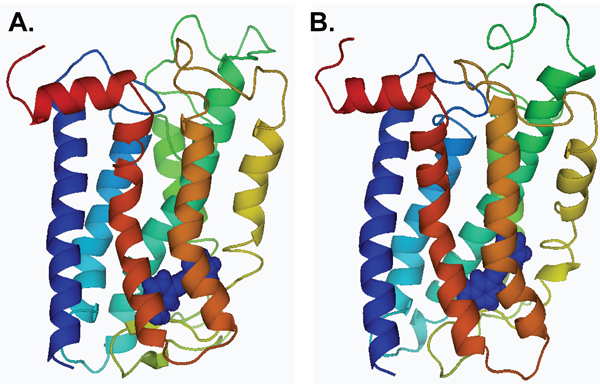
Cartoon representation of the mGluR5 receptor (A) active and (B) inactive models docked with negative modulator MPEP. MPEP is colored in dark blue and is rendered in spheres. MPEP refers to 2-methyl-6-(phenylethynyl)-pyridine. Images were created using Pymol Software [83].

A total of 24 ligands were identified that bind to three mGluR subtypes, the Class I mGluRs mGluR1 and mGluR5, the Class II mGluR, mGluR2 and the Class III mGluRs, mGluR4 and mGluR7. The structures of the ligands are shown in Figure 1. Receptor modulation was reported in human and in rat receptors (for references, see Table 1) and docking was performed with homology models of the mGluR of the respective species. We compared the results from two different docking programs, ArgusLab and AutoDock. Of the rank-ordered list of bound ligand conformations, we have chosen in each case the ligand conformation where the ligand was most buried and had minimum energy in comparison to all other conformations in the same binding pocket. In some cases, ligands were predicted not to bind, but this was only the case for one of the two programs in each case. If a ligand did not dock in AutoDock, it did dock in ArgusLab, and *vice versa*, so that for all ligands binding could be examined. The unbiased searching of the whole receptor with each of the modulators studied revealed that all of the ligands preferentially bound in a region similar to that of retinal in rhodopsin (Figure 2), in the transmembrane domain including helices 3, 5–7 near the interface with the extracellular domain, especially extracellular loop 2. The binding pocket was similar for all ligands docked to all receptors, and is exemplary described in more detail for mGluR5, below.

**Table 1:** List of predicted binding energies for mGluR subtypes 1, 2, 4, 5 and 7 with different positive and negative modulators shown in Figure 1.

					**Binding energies ArgusLab**		**Binding energies AutoDock**	
					
**Class**	**Receptor**	**Modulation**	**Ligand**	**Species**	**Active model [kcal/mol]**	**Inactive model [kcal/mol]**	**Active model [kcal/mol]**	**Inactive model [kcal/mol]**
**I**	**mGluR1**	**Positive**	Ro67-7476 [70]	Rat	-10.02	-9.18	-8.56	-6.88
			Ro01-6128 [70]	Rat	-12.54	-11.06	-7.06	Did not dock
			Ro67-4853 [70]	Rat	-11.16	-10.73	-7.53	Did not dock
		**Negative**	R214127 [71]	Human	-11.53	-12.09	Did not dock	-7.34
			R214127 [71]	Rat	-11.09	-11.97	-9.24	-10.11
			Triaza-fluorenone [72]	Human	Did not dock	-7.81	Did not dock	-6.08 ± 0.15
			CPCCOEt [71]	Rat	-8.60	-9.37	-6.8	-7.46
			YM298198 [73]	Rat	-7.98 ± 0.09	-8.04 ± 0.02	-6.41 ± 0.25	-5.8 ± 0.08
			NPS2390 [72]	Rat	-9.43 ± 0.01	-10.46 ± 0.17	-8.41 ± 0.00	-8.72 ± 0.03
			EM-TBPC [13, 14]	Rat	-8.51	Did not dock	-6.68 ± 0.09	-6.82 ± 0.11
	**mGluR5**	**Negative**	MPEP [74, 75]	Human	-12.83	-13.14	-6.73	-7.77
			DFB-4,4' [16, 76]	Human	-10.47	-11.28	-6.83 ± 0.03	-6.86 ± 0.03
			SIB-1757 [75]	Human	-9.41	-9.74	-6.44	-6.94
			SIB-1893 [75]	Human	-11.71 ± 0.03	-11.82 ± 0.00	-5.83 ± 0.32	-6.63 ± 0.04
			MPEPy [16]	Human	-7.94 ± 0.00	-7.68 ± 0.11	-6.15 ± 0.03	-6.1 ± 0.02
			Fenobam [77]	Human	-7.64	-9.20		
			MTEP [78]	Rat	-9.03	-9.40	-6.2 ± 0.07	-6.21 ± 0.01
		**Neutral**	5MPEP [79]	Rat	-9.52 ± 0.00	-9.41 ± 0.04	-7.04 ± 0.06	-6.66 ± 0.00
		**Positive**	DFB-3,3' [16, 76]	Human	-11.05	-10.06	-7.06	-6.43
			DFB-2,2' [16, 76]	Human	-10.70	-10.02	-6.87 ± 0.04	-6.81 ± 0.01
			CPPHA [76]	Human	-11.38	-9.96	-6.78 ± 0.41	-7.32 ± 0.37
**II**	**mGluR2**	**Positive**	PTEB [80]	Human	-13.94	-12.16	-5.83	-5.1
**III**	**mGluR4**	**Positive**	PHCCC [81]	Human	-9.37 ± 0	-9.31 ± 0.003	-8.07 ± 0.08	-6.16 ± 0.09
	**mGluR7**	**Positive**	AMN082 [82]	Human	-11.27	-13.11	Did not dock	-7.56

**Figure 3:**
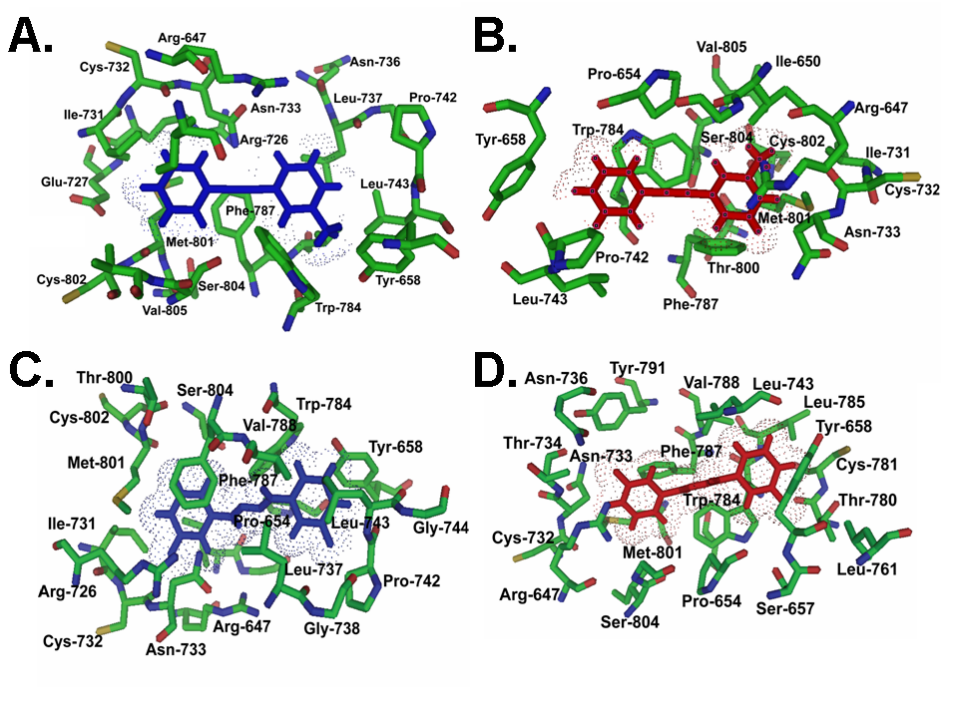
Amino acid residues within 5 Å of the docked ligands MPEP and 3,3'-DFB in mGluR5. The active receptor conformation is shown in A, C and the inactive receptor conformation is shown in B, D. Models were docked with negative modulator MPEP (A, B) and positive modulator 3,3'-DFB (C, D). The ligands are colored in blue for the active models and in red for the inactive models. Images were created using Pymol Software [83].

In order to compare the residues in contact with different ligands, we analyzed the residues predicted to be located within 5 Å distance from the docked ligand. The results obtained with ArgusLab are listed in Table 2 and are shown in Figure 3 for mGluR5 in the active and inactive conformations. To demonstrate the similarities and differences between positive and negative modulators, we compared specifically the negative modulator MPEP with the positive modulator 3,3'-DFB. Analysis of the binding pocket residues in the mGluR5 subtype revealed that W784 was in closest proximity to both docked ligands and in both conformations, active and inactive. W784 is highly conserved in all mGluRs and in class A GPCRs in general. This tryptophan corresponds to W265 in rhodopsin. In addition to W784, residues R647, Y658, L743 and F787 were found to be part of the binding pocket regardless of the type of modulator and conformation of the receptor. In addition, C732, V788, M801 and S804 are found frequently in the binding pockets. In contrast, S657, L785, C781 and T734 were found to be unique for the positive modulator 3,3'-DFB (Figure 3C,D; Table 2) and were not found in the binding pocket of the negative modulator MPEP. Conversely, R726 and V805 were unique to the binding pocket of MPEP (Figure 3A,B; Table 2).

**Table 2:** Residues within 5 Å distance from the MPEP and 3,3'-DFB ligands in active and Inactive models of mGluR5 in comparison to the experimental results published. Residues colored in red – were not predicted in our docking, green – additional residues predicted and black – residues correctly predicted.

	**MPEP Data [14]**	**mGluR5/MPEP Active Model**	**mGluR5/MPEP Inactive Model**	**3,3'-DFB Data [19]**	**mGluR5/3,3'-DFB Active Model**	**mGluR5/3,3'-DFB Inactive Model**
**TM3**	Arg-647, Pro-654, Tyr-658	Arg-647, Ile-650, Tyr-658	Arg-647, Ile-650, Pro-654, Tyr-658	Arg-647, Pro-654, Ser-657, Tyr-658	Arg-647, Pro-654, Tyr-658	Arg-647, Pro-654, Ser-657, Tyr-658
**EC2**	Asn-733	Arg-726, Glu-727, Ile-731, Cys-732, Asn-733, Asn-736	Ile-731, Cys-732, Asn-733	Asn-733	Arg-726, Ile-731, Cys-732, Asn-733	Cys-732, Asn-733, Thr-734, Asn-736
**TM5**	Leu-743	Leu-737, Leu-743, Pro-742	Pro-742, Leu-743	Leu-743	Leu-737, Gly-738, Leu-743, Gly-744, Pro-742	Leu-743
**TM6**	Thr-780, Trp-784, Phe-787, Val-788, Tyr-791	Trp-784, Phe-787, Val-788	Trp-784, Phe-787	Thr-780, Trp-784, Phe-787, Val-788, Tyr-791	Trp-784, Phe-787, Val-788	Thr-780, Trp-784, Phe-787, Cys-781, Leu-785, Val-788, Tyr-791
**TM7**	Met-801, Ala-809	Met-801, Cys-802, Ser-804, Val-805	Thr-800, Met-801, Cys-802, Ser-804, Val-805	Met-801	Thr-800, Met-801, Cys-802, Ser-804	Met-801, Ser-804

### Validation of the ligand binding pocket with experimental data

Site-directed mutagenesis was used previously to identify residues in mGluR5 that are critical for ligand binding [14,19]. Table 2 summarizes the comparison between these experimentally identified ligand binding pocket residues and those predicted by our docking studies with ArgusLab. The results for AutoDock are not shown because the overlap between the predicted binding pockets and those experimentally determined was significantly less. The previous experimental studies with mGluR5 have shown that residues P654, Y658, L743, T780, W784, F787, Y791 and A809 are crucial for binding of the negative modulator MPEP [14]. Our prediction of MPEP binding to both active and inactive GRM5 models predicted all of the above residues to be within 5 Å of the ligand, except A809, T780 and Y791 (colored in red in Table 2). Additionally there are several residues that are predicted to be important for MPEP binding but that have not yet been experimentally verified (colored green in Table 2). For the binding of the positive modulator 3,3'-DFB, it was concluded from site-directed mutagenesis that M801, S657 and T780 are critical for binding and modulatory function [19]. There was also evidence that P654, S657, L743 and N733 may contribute more weakly to 3,3'-DFB binding. All of these residues are predicted to be part of the 3,3'-DFB binding pocket in the inactive model, but S657 and T780 are not present in the active model. In addition, we predict several residues to be part of the binding pockets that have not been investigated previously (shown in green in Table 2). Thus, the comparison of the predicted ligand binding pockets in mGluR5 inactive and active models with the available experimental site directed mutagenesis data strongly validates our models. In addition, we generated testable hypotheses on important residues previously not investigated, and provide evidence that there may be differences in the roles of the amino acids in the binding pocket depending on the conformation of the receptor.

### Analysis of binding energies

**Figure 4:**
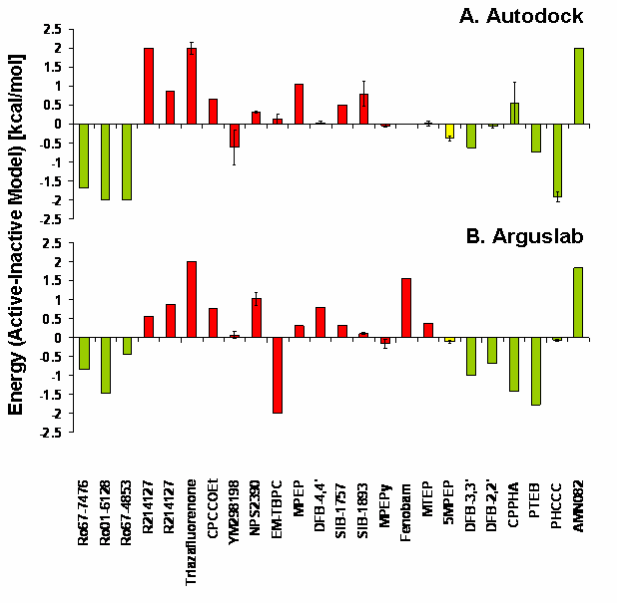
Differences in energy between active (ANM based) and inactive (rhodopsin crystal-structure based) models of mGluRs docked with the ligands shown in Figure 1 and listed in Table 1. Green bars indicate positive modulators, red bars negative modulators and the yellow bar represents a neutral ligand. Where values of 2 are shown, the ligand did not dock to the active model, where values of -2 are shown, the ligand did not dock to the inactive model. Error bars indicate standard deviation in three docking experiments each for the respective active and inactive models. If an error bar is placed at a -2 or 2 bar, the error represents the standard deviation of the ligand and model combination where docking was observed. A. Results from docking with Autodock software. B. Results from docking with ArgusLab software.

The above in-depth analysis of the mGluR5 binding pocket suggests that there may be significant differences between the interactions made by negative and positive modulators with mGluRs depending on the conformation state of the receptor. To test if there is a general trend that distinguishes the action of positive and negative modulators on the receptors, we quantified the overall binding energies for the 24 different ligands with known modulatory nature (positive versus negative). Table 1 shows the binding energies of the ligand-protein complexes calculated by ArgusLab and AutoDock and Figure 4 plots the difference between the respective energies for active and inactive conformations. Where the energies for the active and inactive conformations were very similar, the docking was repeated three times to estimate the error on the predictions (indicated in Table 1 and Figure 4). In general, the results obtained with ArgusLab were less variable between repeated runs than those obtained with AutoDock. A total of 9 ligands were experimentally shown to act as positive modulators of specific subtypes, 14 ligands were negative modulators and one ligand was neutral. In general, the positive modulators bound with more favorable energy to the model of the active mGluR conformation based on the rhodopsin ANM model [18], while the negative modulators bound with more favorable energy to the model of the inactive mGluR conformation based on the rhodopsin inactive, dark-state [17]. The neutral ligand, 5MPEP, showed relatively little differences between the energies of the inactive and the active models, but was consistently better docked to the active model using both programs. ArgusLab predicted 12 of the 14 (86%) negative modulators to bind with more favorable energy to the inactive model and 8/9 (89%) positive modulators to the active model, while the numbers for AutoDock were less correlated: 10/14 (71%) and 7/9 (78%). There were also more incidences in which AutoDock was not able to predict binding for the ligands. Five of the predictions for negative modulators and one positive modulator obtained with AutoDock were near or beyond the accuracy limit of AutoDock as judged by the error obtained when multiple independent docking experiments were carried out. In contrast, in the case of ArgusLab only one difference between docking to active and inactive models was within the noise level. We conclude that the relative difference between the binding energies of the docked ligands for the active and inactive models is highly predictive of the nature of the modulator, positive or negative. Positive modulators in most cases appear to strongly prefer the active conformation over the inactive conformation and negative modulators vice versa.

## Discussion

So far, the only available three-dimensional structure of any GPCR is that of rhodopsin [17]. Previous approaches to docking of ligands to GPCRs have therefore used mostly receptor models based on the rhodopsin structure [13,14,20-43]. The advantages and disadvantages of this approach have been discussed [44,45] and it was shown that in some cases alternative approaches such as the Membstruk modeling approach provides more useful models than those based directly on homology to rhodopsin [46-55]. In particular, it was observed recently that there is a difference in the structures obtained after short molecular dynamics simulations depending on whether receptor agonists or antagonists were docked [56,57]. These observations indicate that the conformation of the receptor will be important for the stability and nature of a receptor-ligand complex. To our knowledge however there has been no previous attempt to predicting different conformations of the receptors first, and then docking ligands to these conformations. We describe here the docking of ligands to two different conformations of mGluR receptors, active and inactive, using two docking programs, ArgusLab [58] and AutoDock [59].

AutoDock is a stochastic Grid-based approach that uses the genetic algorithm to sample different populations of ligand conformations in their binding to the receptor. Each bound conformation is energetically evaluated by a series of energy minimization steps, in which unsuccessful docking results are discarded. While the genetic algorithm is a widely used and reliable algorithm, it has known limitations [60], among the most significant is the possibility of the optimization of the ligand conformations getting trapped in local minima [61]. This is also confirmed by our observation that individual runs may give different results (Figure 4 and Table 1). ArgusLab therefore provides both algorithms, the stochastic search, analogous to the genetic algorithm provided by AutoDock, as well as an exhaustive search method based on identification of complementary shapes of the ligand and the receptor, referred to as "ShapeDock" or "ArgusDock". When using ArgusLab with the ArgusDock algorithm, 12 of the 14 (86%) negative modulators were predicted to bind with more favorable energy to the inactive model. Due to the high reproducibility between different runs, the error margins are small and in all cases but one the errors were significantly smaller than the differences observed between docking to active and inactive models. Similarly, 8/9 (89%) positive modulators bound significantly more favorably to the active model with ArgusLab. The results obtained for AutoDock were less correlated, as expected: 10/14 (71%) and 7/9 (78%) bound the predicted conformation more favorably. We consider the AutoDock results less reliable than those obtained with ArgusLab for several reasons. In six of the predictions, where the differences between docking to active and inactive models were small, docking energy differences were close to or smaller than the noise level. There were also more incidences in which AutoDock was not able to predict binding for the ligands at all. Finally, the ligand binding pockets predicted by AutoDock showed less agreement with the experiments than ArgusLab. However, one should keep in mind that some ligand/binding site types are problematic because the shape does not match if the starting ligand conformation is not correct and/or if the scoring function is not appropriate. This could be the case in the prediction of AMN082, where both algorithms incorrectly predicted this ligand to be a negative modulator, because the ligand did not dock at all to the inactive conformation. Such difficulties may also give rise to larger errors when docking one ligand as compared to another. For example, the docking energies of ligand YM298198 were associated with a larger error when using both algorithms. If we were to equally weigh predictions made by ArgusLab and AutoDock, we would still have agreement between the two methods and strong preferences (i.e. strong differences in both AutoDock and ArgusLab) in 8 out of 14 cases (57%), where the negative modulators bound the inactive conformations with significant preference using both docking programs, and 6 out of 9 cases (67%), supporting the hypothesis.

In addition to comparing predicted ligand binding energies, we also investigated the details of the interactions between the ligand binding pockets and the ligands for mGluR5. Of the experimentally known ligand binding residues of MPEP, our prediction identified all residues, except A809, T780 and Y791 (colored in red in Table 2). These three residues are more than 6.2 Å away from any atom within the ligand, and are not simply missed due to a too short cut-off distance in the definition of ligand binding residues. Furthermore, A809 and T780 are facing the outside of the helical bundle, and it is possible that the experimental effects reported might have been secondary effects. In the case of 3,3'-DFB all known ligand binding residues are also predicted. For both ligands on the other hand, we predict a number of residues to be important for ligand binding that have not been tested experimentally. Finally, we show that while there are several residues in the ligand binding pocket that are shared between active and inactive conformations, there are also residues that bind ligand only in one conformation, and that are specific for positive versus negative modulators. Thus, our models provide useful, experimentally testable hypotheses.

The dependence of the properties of ligand binding on receptor conformation has important functional implications. Recently, it was shown for rhodopsin that the dark-state inactive structure already contains the information needed to form the light-activated structure [18]. This supports the notion that all-trans retinal in rhodopsin stabilizes a conformation that is already partially accessible to the 11-cis retinal bound dark, inactive state of the receptor. Translated to other GPCRs, this suggests that receptors may partially form activated conformations and that agonists could stabilize such conformations, while inverse agonists would destabilize such conformations. In mGluRs, the situation is slightly different from other GPCRs because their ligand binding domain is located in an extracellular domain added to the conserved GPCR seven-transmembrane helical scaffold. In this case, ligands also bind to the transmembrane/extracellular domain interface but here they act as modulators for ligand binding in the extracellular domain. However, it was shown that in the absence of the extracellular domain, positive and negative modulators act as agonists and inverse agonists, respectively. Thus, the findings reported here for mGluRs are likely to have functional implications for the GPCR family in general, implying that agonists and antagonists are likely to prefer the active and inactive conformation of GPCRs, respectively.

## Conclusion

Here we proposed the idea that allosteric ligands can be docked to inactive and active conformational models of mGluRs. We found that the relative difference in binding energy between the two conformations is highly predictive of whether the ligand is a positive or a negative modulator. A positive modulator will bind more favorably to the active conformation, while the negative modulator will bind more favorably to the inactive conformation. Furthermore, we identified similarities and differences in the interactions made between ligand and receptor depending on the nature of the modulator and the conformation of the receptor. The findings are likely to have general utility in predicting functional classification of ligands, such as classification as agonists or antagonists.

## Methods

### Alignment

An alignment of the seven-transmembrane helices of rat and human mGluR1, mGluR2, mGluR4, mGluR5 and mGluR7 with respect to the transmembrane helices of bovine rhodopsin (Protein Data Bank code 1f88[17]) was generated using ClustalW [62]. The alignment was manually validated by comparison with the alignment proposed in previous molecular modeling studies of mGluRs [13,14,63]. Sequences were obtained from SWISS-PROT: mGluR1 (P23385 (rat), Q13255 (human)), mGluR2 (Q14416), mGluR4 (Q14833), mGluR5 (P31424 (rat), P41594 (human)) and mGluR7 (Q14831). The sequence of bovine rhodopsin was read directly from the rhodopsin crystal structure [17].

### Structure prediction using homology modeling

Using the generated sequence alignment, three-dimensional models of the different mGluR subtypes were built by homology modeling using the MODELLER software [64,65]. The crystal structure of dark, inactive bovine rhodopsin with pdb id 1f88[17] and the ANM generated model of the activated state of rhodopsin [18] were used as the structural templates for generating the inactive and active models of mGluRs, respectively. All models were evaluated using PROCHECK [66], MOLPROBITY [67] and WHAT-IF [68].

### Docking with ArgusDock

We assembled from literature a list of ligands for which their effect (positive or negative modulation) is known (listed in Table 1). All ligands were docked to inactive and active models of the respective mGluR subtypes using ArgusLab software, version 4.0 [58]. Ligand pdb files were generated using JME Molecular Editor software [69]. Hydrogen atoms were added to the ligand coordinate file prior to docking using ArgusLab. The docking between each receptor subtype and ligand was performed using the "Dock a ligand" option. All the residues of the receptor were defined to be part of the binding site i.e, cubic boxes measuring 151 × 123 × 145 points for the inactive model and 111 × 151 × 151 for the active model were built to include the entire protein in each case, allowing no bias towards the binding pocket. A spacing of 0.4 Å between the grid points was used. Docking simulations were performed by selecting "ArgusDock" as the docking engine. "Dock" was chosen as the calculation type, "flexible" for the ligand and the AScore was used as the scoring function. The AScore function, with the parameters read from the AScore.prm file was used to calculate the binding energies of the resulting docked structures. This file contains the coefficients for each term in the scoring function. Structures were visualized and the best docked structure was chosen based on lowest energy and minimal solvent accessibility of the ligand, as follows. First, the top 150 unique poses were retrieved. Typically, 20% of these conformations were ligands docked to the surface of the protein, highly water accessible. These conformations were discarded manually. In at least ~2/3 of the remaining structures, the ligand was bound in a pocket analogous to the retinal binding pocket. Those ligands that were only partially buried in the protein interior, with parts of the ligand facing the outside of the helical bundle, were also discarded. Only ligands with maximal burial in the protein interior were retained. This typically included a list of 50 structures. These structures were rank-ordered by minimum energy and the structure with the lowest energy was chosen as the predicted receptor-bound conformation of the ligand.

### Docking with AutoDock

All ligands shown in Figure 1 were docked to inactive and active models of the various mGluR subtypes, using the Lamarckian Genetic algorithm (LGA) provided by the AutoDock program, version 3.0 [59]. Solvation parameters were added to the protein coordinate file with the "Addsol" option in AutoDock, and the ligand torsions were defined using the "Ligand torsions" menu option of AutoDock. The grid maps representing the protein were calculated using the "AutoGrid" option. A cubic box was built around the protein with 126 × 126 × 126 points; a spacing of 0.403 Å between the grid points was used. The protein was centered on the geometric center prior to docking. Docking simulations were carried out with an initial population of 300 individuals, and a maximum number of 50,000,000 energy evaluations. Apart from this a maximum number of 27,000 generations, a translation step of 2 Å, a quarternion step of 50° and a torsion step of 50° were used as the docking parameters for obtaining the final docked structures. Resulting orientations that have less than or equal to 0.5 Å root mean square deviation were clustered. In addition to returning the docked structure, AutoDock also calculates an affinity constant for each ligand-receptor configuration. The best ligand-receptor structure from the docked structures was chosen based on lowest energy and minimal solvent accessibility of the ligand, analogous to the procedure described above for ArgusLab, with the difference being that only the top 10 most favorably bound ligand structures were analyzed.

## List of abbreviations

**mGluR**, metabotropic glutamate receptor; **GPCR**, G protein coupled receptor; **CPCCOEt**, cyclopropan[*b*]chromen-1*a*-carboxylate; **DFB**, difluorobenzaldazine; **fenobam**, *N*-(3-chlorophenyl)-*N*'-(4,5-dihydrol-1-methyl-4-oxo-1-*H*-imidazole-2-yl)-urea; **MPEP**, 2-methyl-6-((3-methoxyphenyl)ethynyl)-pyridine; **R214127**, 1-(3,4-dihydro-2*H*-pyrano[2,3-*b*]quinolin-7-yl)-2-phenyl-1-ethanone; **Ro01-6128**, diphenylacetyl-carbamic acid ethyl ester; **Ro67-4853**, (9*H*-xanthene-9-carbonyl)-carbamic acid butyl ester; **Ro67-7476**, (*S*)-2-(4-fluoro-phenyl)-1-(toluene-4-sulphonyl)-pyrrolidine; **SIB1757**, 6-methyl-2-(phenylazo)-3-pyrindol; **SIB1893**, ([phenylazo]-3-pyrindole)-2-methyl-6-(2-phenylethenyl)pyridine; **MTEP**, 3-[(2-methyl-1,3-thiazol-4-yl)ethynyl]pyridine; **YM298198**, (6-([(2-methoxyethyl)amino]methyl)-*N*-methyl-*N*-neopentylthiaolo[3,2-a]benzoimidazole-2-carboxamide; **EM-TBPC **– 1-ethyl-2-methyl-6-oxo-4-(1,2,4,5-tetrahydro-benzo[d]azepin-3-yl)-1,6-dihydro-pyrimidine-5-carbonitrile; **PTBE**, (1-(2-hydroxy-3-propyl-4,4-[4-(2*H*-tetrazol-5-yl)phenoxy]butoxyphenyl)ethanone); **NPS2390**, 2-quinoxaline-carboxamide-*N*-adamantan-1-yl; **CPPHA**, *N*-(4-chloro-2-[(1,3-dioxo-1,3-dihydro-2*H*-isoindol-2-yl)methyl]phenyl)-2-hydroxybenzamide; **5MPEP**, 5-methyl-6-(phenylethynyl)-pyridine; **MPEPy**, 3-Methoxy-5-pyridin-2-ylethynylpyridine; **PHCCC**, *N*-phenyl-7-(hydroxylimino)cyclopropa[*b*]chromen-1a-carboxamide; **AMN082**, N,N'-Dibenzhydrylethane-1,2-diamine dihydrochloride.

## Authors' contributions

NY carried out all the modeling and docking studies, analyzed and interpreted the data and wrote the manuscript. KT collected the list of ligands and their effects on mGluRs and participated in the analysis of the docked structures. JKS designed the hypothesis and computational experiments to test it, participated in analysis and interpretation of data and the writing of the manuscript.

## Competing interests

The authors declare that they have no competing interests.
